# 6-Thioguanine-loaded polymeric micelles deplete myeloid-derived suppressor cells and enhance the efficacy of T cell immunotherapy in tumor-bearing mice

**DOI:** 10.1007/s00262-015-1702-8

**Published:** 2015-05-16

**Authors:** Laura Jeanbart, Iraklis C. Kourtis, André J. van der Vlies, Melody A. Swartz, Jeffrey A. Hubbell

**Affiliations:** 1 Institute of Bioengineering, School of Life Sciences and School of Engineering, Ecole Polytechnique Fédérale de Lausanne (EPFL), Lausanne, Switzerland; 2 Swiss Institute for Experimental Cancer Research (ISREC), School of Life Sciences, EPFL, Lausanne, Switzerland; 3 Institute for Chemical Sciences and Engineering, School of Basic Sciences, EPFL, Lausanne, Switzerland; 4 Institute for Molecular Engineering, University of Chicago, Chicago, IL USA; 5 Materials Science Division, Argonne National Laboratory, Argonne, IL USA

**Keywords:** MDSC depletion, 6-Thioguanine, Cancer, T cell therapy

## Abstract

Myeloid-derived suppressor cells (MDSCs) are a heterogeneous population of immature myeloid cells that suppress effector T cell responses and can reduce the efficacy of cancer immunotherapies. We previously showed that ultra-small polymer nanoparticles efficiently drain to the lymphatics after intradermal injection and target antigen-presenting cells, including Ly6c^hi^ Ly6g^−^ monocytic MDSCs (Mo-MDSCs), in skin-draining lymph nodes (LNs) and spleen. Here, we developed ultra-small polymer micelles loaded with 6-thioguanine (MC-TG), a cytotoxic drug used in the treatment of myelogenous leukemia, with the aim of killing Mo-MDSCs in tumor-bearing mice and thus enhancing T cell-mediated anti-tumor responses. We found that 2 days post-injection in tumor-bearing mice (B16-F10 melanoma or E.G7-OVA thymoma), MC-TG depleted Mo-MDSCs in the spleen, Ly6c^lo^ Ly6g^+^ granulocytic MDSCs (G-MDSCs) in the draining LNs, and Gr1^int^ Mo-MDSCs in the tumor. In both tumor models, MC-TG decreased the numbers of circulating Mo- and G-MDSCs, as well as of Ly6c^hi^ macrophages, for up to 7 days following a single administration. MDSC depletion was dose dependent and more effective with MC-TG than with equal doses of free TG. Finally, we tested whether this MDSC-depleting strategy might enhance cancer immunotherapies in the B16-F10 melanoma model. We found that MC-TG significantly improved the efficacy of adoptively transferred, OVA-specific CD8^+^ T cells in melanoma cells expressing OVA. These findings highlight the capacity of MC-TG in depleting MDSCs in the tumor microenvironment and show promise in promoting anti-tumor immunity when used in combination with T cell immunotherapies.

**Electronic supplementary material:**

The online version of this article (doi:10.1007/s00262-015-1702-8) contains supplementary material, which is available to authorized users.

## Introduction

Over the past decades, many novel cancer immunotherapies have been developed to boost anti-tumor immunity, targeting a variety of mechanisms including tumor antigen presentation by dendritic cells (DCs), anti-tumor T cell priming, overall T cell activation status, immune suppression, and T cell infiltration in the tumor [1, 2]. Strategies have included cell-based therapies such as transfer of ex vivo activated DCs or engineered T cells as well as antibody-based therapies that target specific T cell inhibitory pathways including CTLA-4 or PD-1/PD-L1 [3–6]. Despite these efforts, many therapeutic modalities encounter limited success because of tumor-induced immune suppression and evasion mechanisms [6–9]. It has been shown that targeting these immune suppressive mechanisms can lead to enhanced immunotherapy efficacy in cancer [10–14].

Myeloid-derived suppressor cells (MDSCs) are a heterogeneous population of immature myeloid cells, characterized by their expression of CD11b and Gr1 and lack of MHCII; they comprise a Ly6c^hi^ Ly6g^−^ Gr1^int^ monocytic subset (Mo-MDSCs) and a Ly6c^lo^ Ly6g^+^ Gr1^hi^ granulocytic subset (G-MDSCs) [15]. MDSCs are induced by tumor-mediated inflammation [16–18], recruited to the circulation via tumor-derived factors such as IL-1, IL-6, GM-CSF, G-CSF, and VEGF [19–21], and accumulate in the tumor, tumor-draining lymph node (LN), and spleen, with MDSC numbers increasing with tumor load [16, 21]. MDSCs play a major role in anti-tumor immunity by inhibiting both CD8^+^ and CD4^+^ T cell activation, proliferation, and homing [16, 17, 22, 23]. G-MDSCs infiltrate and exert their suppressive activity in an antigen-specific manner in the LNs, while Mo-MDSCs, considered as the more suppressive ones, infiltrate and suppress T cell responses in the spleen and tumor [18, 24–26].

Strategies to target MDSCs, and thereby improve local T cell function, include depletion (affecting both recruitment and expansion in the tumor), functional inhibition, and differentiation into mature antigen-presenting cells [27, 28]. Ly6c^hi^ monocytes and Mo-MDSCs traffic from the bone marrow (BM) to sites of inflammation via CCR2-signaling [29], and therapeutic strategies based on CCR2-siRNA showed significant reduction in inflammatory monocyte effects in murine models of atherosclerosis, cancer, and diabetes [30]. Also, all-trans-retinoic acid (RA), which is important for hematopoietic stem cell development [31], was shown to eliminate immature myeloid cells in tumor-bearing mice and drive their differentiation into mature myeloid cells in cancer patients [10, 32, 33], although without affecting tumor growth. The chemotherapeutic pyrimidine analogs gemcitabine and 5-fluorouracil, which prevent DNA replication and lead to apoptosis, have shown efficacy in depleting MDSCs in tumors and lymphoid organs, leading to expansion of tumor-specific T cells and delayed tumor growth in mice [34, 35]. High doses of the TLR9 agonist CpG have been shown to impact both Ly6g^+^ and Ly6c^hi^ MDSCs in tumor-bearing mice by decreasing their suppressive function and leading to their differentiation into mature myeloid cells [36, 37]. Finally, combining adoptive T cell therapy or DC transfer with cytotoxic drugs or small molecule inhibitors has shown some promise in slowing tumor growth [38–41].

The purine analog 6-thioguanine (TG) is an effective anti-inflammatory and anticancer drug [42]. It is used in pediatric and adult leukemias, including myeloid and myelogenous leukemias [43, 44], where it can “freeze” myeloblasts in an immature state to prevent their differentiation into mature myeloid cells, including monocytes and granulocytes [18, 45]. We hypothesized that TG might be an interesting drug candidate to deplete Mo-MDSCs based on its ability to target the myeloid cell lineage.

MDSC-directed strategies have been shown to enhance anti-tumor immunity and to synergize with anticancer vaccines [10, 11, 40]. Our laboratory has developed nanoparticles (NPs) that drain from i.d. administration sites through lymphatics to target skin-draining LNs, where they are taken up by resident antigen-presenting cells [46, 47]. In a biodistribution study, we showed that NPs are taken up remarkably effectively by Mo-MDSCs in LNs, spleen, and tumor [47]. Based on this observation, we developed a nanoscale polymeric micelle (MC) capable of sequestering and releasing TG, with the intention of depleting MDSCs in a more targeted manner than might be achievable with free TG. MCs consist of TG chemically conjugated via a disulfide bond to a block copolymer of the hydrophilic polyethylene glycol (PEG) and the hydrophobic polypropylene sulfide (PPS) [48]. After first finding that MC-TG entirely depleted BM-derived Mo-MDSCs, we then tested the effect of MC-TG on MDSCs in two different tumor models in mice and assessed the efficacy of the drug in micellar compared to soluble form. Finally, we combined our MDSC-targeting strategy with two modes of cancer immunotherapy and demonstrated that MC-TG enhances the efficacy of adoptive T cell immunotherapy.

## Materials and methods

### Mice and cell lines

C57BL/6 female mice, aged 8–12 weeks, were obtained from Harlan (France) and OT-I mice, C57BL/6-Tg(TcraTcrb)1100Mjb/J, from Charles River Laboratories (France). All experiments were performed with approval from the Veterinary Authority of the Canton de Vaud (Switzerland) according to Swiss Law.

E.G7-OVA thymoma cells (OVA-expressing EL-4 cells, ATCC CRL-2113) and B16-F10 melanoma cells (ATCC CRL-6475) were obtained from American Type Culture Collection (Manassas, VA, USA). Ovalbumin-expressing B16-F10 cells (B16.OVA) were a kind gift of Bertrand Huard (University Medical Center, Geneva, Switzerland). E.G7-OVA cells were cultured in RPMI 1640 medium supplemented with 10 % FBS, 10 mM HEPES, 1 mM sodium pyruvate (all from Life Technologies, Carlsbad, CA, USA), 0.05 mM β-mercaptoethanol, and 0.4 mg/ml G418 (Brunschwig, Basel, Switzerland); they were expanded in G418-free media just before inoculation. B16-F10 cells were maintained in DMEM supplemented with 10 % FBS.

### Reagents

Chemicals, including 6-thioguanine, were reagent grade and purchased from Sigma-Aldrich (Saint Louis, MO, USA). 5′ SPO_3_-CpG oligonucleotide (5′-TCCATGACGTTCCTGACGTT-3′) was purchased from Microsynth (Balgach, Switzerland). Low-endotoxin-grade OVA (<0.01 EU/μg protein), used for immunization, was from Hyglos (Bernried, Germany), and OVA grade VI, used for restimulation, was purchased from Sigma-Aldrich. IL-6 and GM-CSF were purchased from Peprotech (Oak Park, CA, USA).

### Nanoparticle synthesis and formulation

#### NP-OVA and NP-CpG

Pluronic-stabilized PPS NPs were synthesized by emulsion polymerization and surface functionalized as previously described [49]. Before and after conjugation, the size of NPs was determined by dynamic light scattering (DLS) (Zetasizer, Nano ZS, Malvern Instruments, Malvern, UK) and was approximately 30 nm. OVA and CpG were conjugated to NPs as previously described [50]. Concentrations of OVA and CpG on NPs were determined by Pierce BCA protein assay (Perbio Thermo Fischer Scientific, Waltham, MA, USA) and by GelRed assay (Brunschwig, Basel, Switzerland), respectively. All NP formulations displayed endotoxin levels lower than 0.1 EU per dose administered to mice, as detected using the HEK-Blue hTLR4 cells from Invivogen (San Diego, CA, USA).

#### MC-TG

MC-TG was formed as previously described [48]. Briefly, PEG–PPS–SS–TG was dissolved by vortexing and gentle heating in *N*-methyl-2-pyrrolidone to 100 mg/ml. The pale yellow solution was added dropwise to stirred endotoxin-free water at a 1:9 volume ratio (polymer/water) and stirred for 10 min. The mixture was then transferred to a 3500 MWCO cellulose membrane (Spectrum Laboratories, Rancho Dominguez, CA, USA) and dialyzed against water overnight. Formed MCs were then collected, filtered (0.22 μm), and concentrated in 3000 MWCO Amicon filter tubes according to manufacturer’s instructions (Millipore, Billerica, MA, USA). The concentration of TG was measured by UV/Vis at 340 nm by first releasing TG by TCEP reduction. Formed MCs (MC-TG) had a concentration of 10–12 μM TG and a diameter of 25 nm by DLS (supplementary Fig. S1).

### In vitro assays of MDSCs

#### Culture of BM-derived MDSCs

BM-derived MDSCs were cultured as reported [51]. BM from C57BL/6 femurs and tibias were collected in RPMI 1640 medium supplemented with 10 % FBS, 1 % penicillin/streptomycin, 10 mM HEPES, and 20 μM β-mercaptoethanol (MDSC media). Cells were filtered through a 70-μm strainer, and red blood cells (RBCs) were lysed 5 min at room temperature with NH_4_Cl. Cells were plated at a density of 164,000 cells/ml in 12-well plates or at 250,000 cells/ml in petri dishes with 40 ng/ml IL-6 and 40 ng/ml GM-CSF. Cells were incubated 4 days and then collected for analysis or replated and incubated for another 3 days.

#### OVA-specific CD8^+^ T cell proliferation assay

2 × 10^5^ OT-I splenocytes were plated in 96-well plates and co-cultured with BM-derived MDSCs at varying concentrations (2 × 10^5^ MDSCs correspond to 100 % MDSCs to splenocytes, and no MDSCs correspond to 0 % MDSCs to splenocytes). Cells were cultured in MDSC medium and incubated 24 h with 250 μg/ml OVA grade VI. 0.5 μCi of ^3^H thymidine was added to each well, and cells were further incubated for 18 h. Cells were then stored at −20 °C before collection on filter plates and analysis by a scintillation counter to determine thymidine incorporation.

### Tumor inoculation and injections

Mice were anesthetized with isoflurane (5 % for induction and 2 % for maintenance) and injected with 10^6^ E.G7-OVA cells, 5 × 10^5^ B16-F10 cells, or 2.5 × 10^5^ B16.OVA cells in 30 μl 0.9 % saline solution intradermally (i.d.) on the left side of the back. E.G7-OVA, B16-F10, or B16.OVA tumor-bearing mice were injected 7, 5, or 4 days post-tumor inoculation (p.i.), respectively, with 10 mg/kg MC-TG or free TG injected i.d. in all four footpads (unless otherwise specified) in the following experiments:

- *Biodistribution* MC-TG was labeled with the fluorophore Dy649; mice were killed on day 9;
- *Time course* blood was sampled every 2–3 days starting on injection day;
- *Multiple doses* mice were boosted on day 13 with 5 mg/kg MC-TG;
- *Dosage* mice were injected with 2, 5, or 10 mg/kg MC-TG on day 7 and killed on day 14;
- *NP*-*vaccine* mice were immunized on days 3 and 10 with 10 μg NP-OVA and 1 μg NP-CpG (NP-vaccine) i.d. in the front footpad draining the tumor; mice were injected with 10 mg/kg MC-TG on day 13;
- *Adoptive T cell transfer* 10 mg/kg MC-TG or free TG was injected i.d. on day 4 p.i., and 2 days later (day 6 p.i.), 10^6^ OT-I CD8^+^ T cells were transferred i.v. in the tail vein.

Blood was sampled from the submandibular vein of the cheek with a 4-mm lancet at indicated time points. Tumors were measured starting 5 days p.i. with a digital caliper, and volumes (*V*) were calculated as an ellipsoid (*V* = *π*/6 · l ·*w* · *h*, where *l* is length, w width, and *h* height). Mice were killed by CO_2_ asphyxiation. Experiments were stopped when tumor volumes reached 1 cm^3^ or earlier if necrotic.

### Adoptive CD8^+^ T cell transfer

Splenic CD8^+^ T cells from OT-I mice cells were isolated by immunomagnetic negative selection (EasySep Mouse CD8^+^ T Cell Isolation Kit) and CD11c^+^ by positive selection (EasySep Mouse CD11c Positive Selection Kit), both from Stemcell Technologies (Vancouver, BC, Canada). CD8^+^ and CD11c^+^ cells were co-cultured 72 h at a ratio of 10:1 with 1 nM OVA_257-264_ peptide (Genscript, Piscataway, NJ, USA) and 10 U/ml recombinant mouse IL-2 (Roche, Rotkreuz, Switzerland). Cells were then collected, washed in basal medium, and resuspended to 10^7^ cells/ml prior to tail vein injection.

### Tissue and cell preparation

Spleens, LNs (brachial, axillary, inguinal), and tumors were harvested at time of killing. LNs and tumors were digested 20 and 60 min, respectively, in DMEM supplemented with 1 mg/ml collagenase D (Roche). Single-cell suspensions were obtained by gently disrupting the organs through a 70-μm cell strainer. Spleen and blood RBCs were lysed with NH_4_Cl 5 min. Cells were counted and resuspended in IMDM supplemented with 10 % FBS and 1 % penicillin/streptomycin (full medium) (all from Life Technologies).

### Flow cytometry

Cells were washed and stained with surface antibodies in staining buffer [HBSS (Life Technologies) supplemented with 0.5 % bovine serum albumin]. Cell viability was determined by propidium iodide incorporation in staining buffer after surface antibody staining or with live/dead fixable cell viability reagent (Life Technologies) in PBS before antibody staining. Cells were stained with PE-labeled H-2Kb/OVA_257–264_ pentamer (Proimmune, Oxford, UK) according to manufacturer’s instructions.

AccuCount cell counting beads (Spherotech, Lake Forest, IL, USA) were added to blood samples. Samples were acquired on CyAn ADP analyzer (Beckman Coulter, Brea, CA, USA), and data were analyzed with FlowJo software (v9.4; Tree Star, Ashland, OR, USA). Antibodies against mouse CD8, CD3, MHCII, B220, CD45, CD11b, Gr1, Ly6c, Ly6g, and CD11c were purchased from eBioscience or BioLegend (San Diego, CA, USA). Pacific orange-conjugated and Alexa Fluor 647-conjugated streptavidins were from Life Technologies.

### Statistical analysis

Statistically significant differences between experimental groups were determined by one-way analysis of variance (ANOVA) followed by Bonferroni posttest correction with Prism software (v5, GraphPad, San Diego, CA, USA). *, **, and *** indicate *P* values <0.05, 0.01, and 0.001, respectively.

## Results

### MC-TG depletes BM-derived Mo-MDSCs in vitro

Based on the hypothesis that TG in a nanoparticulate formulation may be more readily targeted to MDSCs than in soluble form [47], we formulated TG as a 25-nm micelle (MC-TG) by linking TG to a PEG–PPS chain via a disulfide bond (supplementary Fig. S1) [48]. We generated MDSCs in vitro following a well-established protocol [51] (supplementary Fig. S2 A) using IL-6 and GM-CSF, two factors secreted by tumors that recruit MDSCs from the BM to the circulation in tumor-bearing mice [19–21]. After 4 days of culture, BM cells were skewed toward a CD11b^+^ MHCII^−^ CD11c^−^ immature myeloid phenotype characteristic of MDSCs, with Ly6c^hi^ Ly6g^−^ Mo-MDSC and Ly6c^lo^ Ly6g^+^ G-MDSC subsets (supplementary Fig. S2 B) [16].

**Fig. 1:**
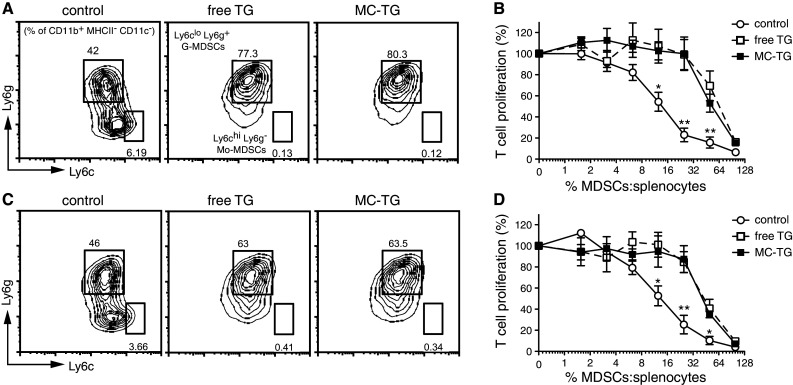
MC-TG depletes BM-derived Mo-MDSCs in vitro. **a**, **b** BM cells were incubated for 4 days in media conditioned with 1 μM free TG or MC-TG, after which cells were analyzed by flow cytometry or co-cultured with OT-I splenocytes and OVA: **a** representative flow cytometry plots of CD11b^+^ MHCII^−^ CD11c^−^ immature myeloid cells stained for Ly6c and Ly6g (*values* in the *dot plots* represent percentage of CD11b^+^ MHCII^−^ CD11c^−^ cells in each gate) (average values are given in the text); **b** OT-I T cell proliferation as determined by ^3^H thymidine incorporation. **c**, **d** BM cells were incubated for 4 days to allow MDSC differentiation, after which they were replated in medium conditioned with 1 μM free TG or MC-TG and incubated for 3 days: **c** representative flow cytometry plots of immature myeloid cells stained for Ly6c and Ly6g (*values* in the *dot plots* represent percentages of CD11b^+^ MHCII^−^ CD11c^−^ cells in each gate; average values are given in the text); **d** OT-I T cell proliferation as determined by ^3^H thymidine incorporation

TG, in both free and micellar forms, depleted Mo-MDSCs in vitro (Fig. 1a). Mo-MDSCs were reduced from 5.6 ± 0.5 % of the culture to 0.1 % (**) of the culture with free TG and MC-TG. While control MDSCs efficiently prevented OT-I T cell proliferation, adding either free TG or MC-TG to MDSCs rendered them less suppressive (Fig. 1b). Furthermore, both MC-TG and free TG depleted already differentiated Mo-MDSCs by day 7 in vitro (Fig. 1c), from 3.9 ± 0.7 to 0.2 and 0.3 %, respectively, and rendered BM-derived MDSCs less suppressive than control MDSCs (Fig. 1d). While a ratio of approximately 1:8 of control MDSCs/splenocytes (12 %) was needed to achieve a 50 % reduction in T cell proliferation, approximately 40 % TG-treated MDSCs were needed to achieve equivalent inhibition of T cell proliferation (Fig. 1d). These results show that TG, in both free and micellar forms, can deplete BM-derived Mo-MDSCs in vitro and can render MDSCs overall less suppressive against T cell proliferation.

### MC-TG targets and depletes MDSCs in the spleen, LNs, and tumor after 2 days

**Fig. 2:**
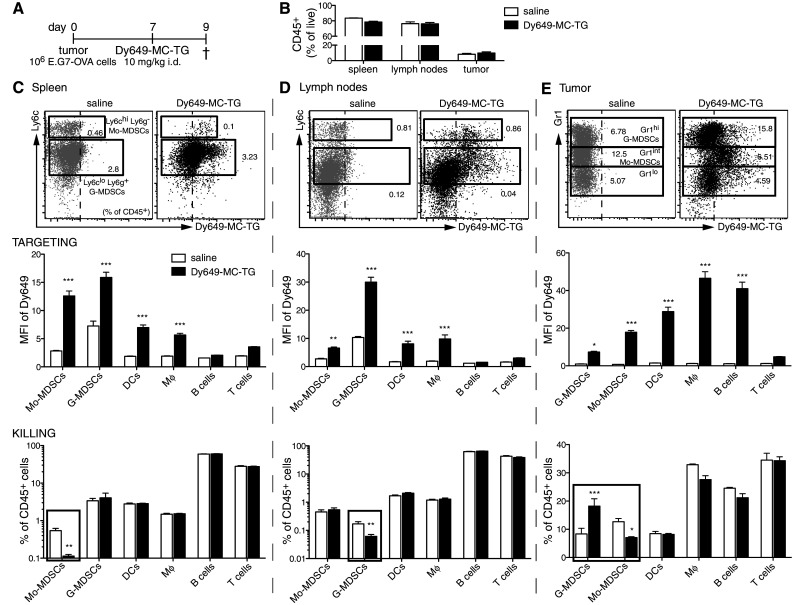
MC-TG targets and depletes MDSCs in the spleen, LNs, and tumor after 2 days. **a** Injection timeline of E.G7-OVA tumor-bearing mice injected 7 days p.i. with 10 mg/kg of fluorescently labeled MC-TG (with Dy649) i.d. in the four footpads. Mice were killed 2 days later: Spleen, tumor, and LNs (axillary, brachial, inguinal) were collected and analyzed by flow cytometry. **b** Frequency of CD45^+^ leukocytes in spleen, LNs, and tumor (as  % of live cells). **c** Spleen, **d** LNs, **e** tumor: *upper* representative flow cytometry plots of MDSCs: Ly6c (spleen, LNs) or Gr1 (tumor) staining versus fluorescence of Dy649 (*values* in the *dot plots* represent percentage of CD45^+^ cells in each gate); *middle* mean fluorescence intensity (MFI) in Dy649 channel of Mo-MDSCs, G-MDSCs, DCs, Mφ, B cells, T cells; *lower* proportion of Mo-MDSCs, G-MDSCs, DCs, Mφ, B cells, T cells as percentage of CD45^+^ cells. Four mice per group, ***P < 0.001, **P < 0.01, *P < 0.05. [MDSCs defined as CD11b^+^ MHCII^−^ CD11c^−^, DCs as CD11c^+^ MHCII^+^, Mφ as CD11b^+^ MHCII^+^, B cells as B220^+^, T cells as CD3^+^]

Next, we investigated the effects of MC-TG on MDSCs in vivo. E.G7-OVA tumor-bearing mice were injected with fluorescently labeled MC-TG (for tracking purposes) 7 days p.i. (Fig. 2a). MC-TG did not affect frequencies of CD45^+^ cells (Fig. 2b) and was efficiently taken up by both Mo-MDSCs and G-MDSCs in the spleen and LNs of tumor-bearing mice 2 days post-injection (Fig. 2c, d *upper*). Spleen DCs and macrophages (Mφ), but not B or T cells, also took up MC-TG (Fig. 2c, d *middle*). After 2 days, MC-TG selectively decreased frequencies of Mo-MDSCs in the spleen and G-MDSCs in LNs, leaving other targeted cells unaffected at this time point (Fig. 2c, d *lower*). All tumor MDSC subsets took up MC-TG (Fig. 2e *upper*), and MC-TG also associated with tumor-associated DCs, Mφ, and B cells (Fig. 2e *middle*). MC-TG significantly decreased the frequency of tumor-infiltrating Gr1^int^ Mo-MDSCs and increased Gr1^hi^ G-MDSCs, but did not affect other targeted cells (Fig. 2e *lower*). Taken together, these results show that MC-TG acts on Mo-MDSCs in the spleen, G-MDSCs in the LNs, and Gr1^int^ Mo-MDSCs in the tumor. MC-TG and TG seemed well tolerated.

### MC-TG depletes circulating Mo-MDSCs and G-MDSCs in tumor-bearing mice

**Fig. 3:**
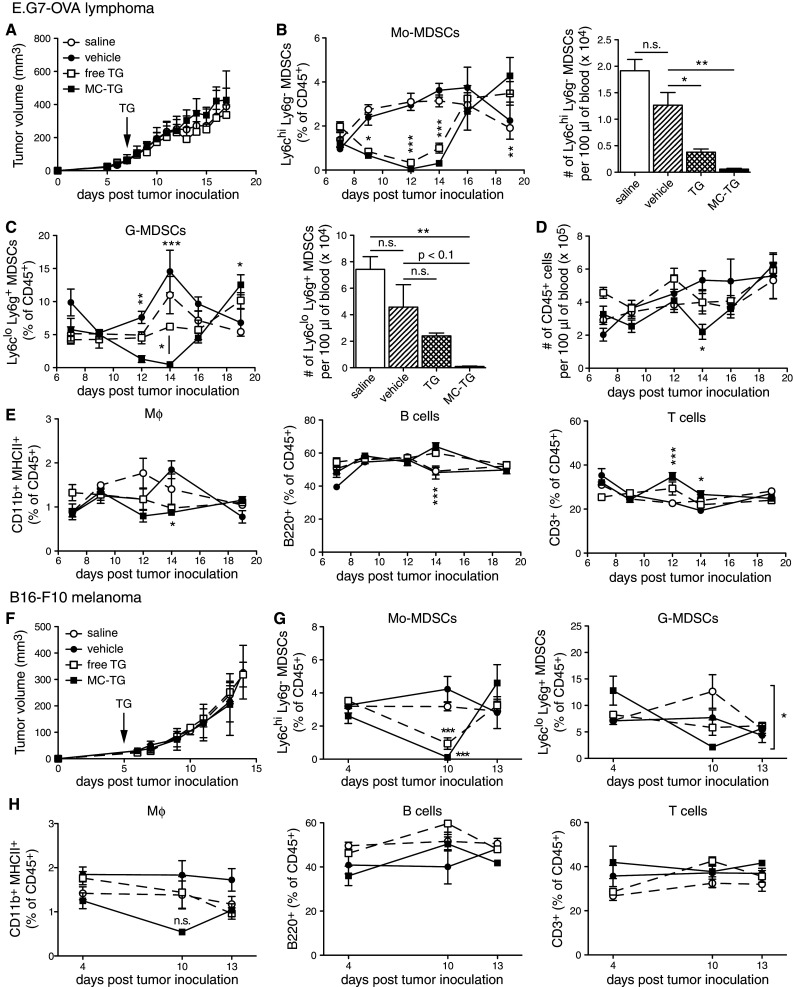
MC-TG depletes circulating Mo-MDSCs and G-MDSCs and Mφ in tumor-bearing mice. **a**–**e** E.G7-OVA tumor-bearing mice were injected 7 days p.i. with 10 mg/kg TG i.d. in the four footpads. **a** E.G7-OVA tumor volumes. Proportions over time (as percentage of CD45^+^ cells, *left*) and numbers on day 14 (per 100 μl blood, *right*) of circulating (**b**) Mo-MDSCs and (**c**) G-MDSCs. **d** Number of CD45^+^ leukocytes per 100 μl blood over time. **e** Proportions of Mφ, B cells, and T cells (as percentage of CD45^+^ cells) in blood over time. **f**–**h** B16-F10 tumor-bearing mice were injected 5 days p.i. with 10 mg/kg TG i.d. in all four footpads. **f** B16-F10 tumor volumes. Proportion of (**g**) Mo-MDSCs (*left*) and G-MDSCs (*right*), and of (**h**) Mφ, B cells, and T cells in the blood over time as percentage of CD45^+^ cells. Experiments repeated, 4–5 mice per group. ***P < 0.001, **P < 0.01, *P < 0.05, *n.s.* not significant. [MDSCs defined as CD11b^+^ MHCII^−^]

Next, we sought to characterize the temporal effects of free TG and MC-TG. While one dose of free or MC-TG did not impact E.G7-OVA tumor growth (Fig. 3a), MC-TG significantly reduced frequencies of Mo-MDSCs and G-MDSCs in the blood, with almost no Mo-MDSCs and G-MDSCs remaining 7 days post-MC-TG injection (Fig. 3b, c), which also corresponded to a reduction in total leukocytes on day 14 (Fig. 3d). Free TG also decreased Mo-MDSCs, but did not entirely deplete them as did MC-TG. Free TG or vehicle control did not affect G-MDSC frequencies (Fig. 3c). Interestingly, both MDSC subsets repopulated the blood compartment on day 16 and even surpassed steady-state values by day 18 p.i.. CD11b^+^ MHCII^+^ Mφ were left unaffected by TG except on day 14 (Fig. 3e), when both Ly6c^hi^ CD11b^+^ MHCII^+^ Mφ (monocytes) and Ly6g^+^ CD11b^+^ MHCII^+^ Mφ (neutrophils) were substantially reduced and Ly6c^lo/−^ Ly6g^−^ Mφ were left unaffected (supplementary Fig. S3 A-C). MC-TG and soluble TG had no impact on frequencies of circulating B and T cells (Fig. 3e).

The above experiment was reproduced in the B16-F10 melanoma model, which is an orthotopic, more immunosuppressive, and more aggressive cancer model [52]. As in the E.G7-OVA model, MC-TG did not affect B16-F10 tumor growth (Fig. 3f) and depleted circulating Mo-MDSCs 5 days post-injection (Fig. 3g *left*); free TG also reduced Mo-MDSCs but did not deplete them. G-MDSCs levels, not affected by free TG, were significantly reduced by MC-TG (Fig. 3g *right*). Mφ, B, and T cells were not affected by MC-TG (Fig. 3h). Together, these results show that a single injection of MC-TG depleted Mo-MDSCs and G-MDSCs for 7 days in both E.G7-OVA and B16-F10 cancer models. MC-TG also depleted circulating monocytic Ly6c^hi^ Mφ (CD11b^+^ MHCII^+^ mature myeloid cells). Finally, MC-TG was more effective than free TG in depleting MDSCs without affecting tumor growth.

### Dose and schedule of MC-TG delivery modulate MDSC depletion

**Fig. 4:**
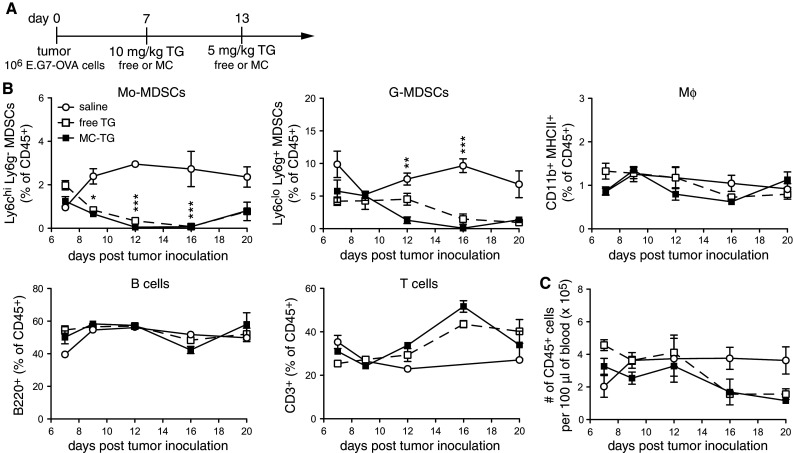
Two doses of MC-TG deplete circulating MDSCs over 2 weeks. **a** Injection timeline of E.G7-OVA tumor-bearing mice injected 7 days p.i. with 10 mg/kg MC-TG i.d. in the four footpads and boosted on day 13 with 5 mg/kg MC-TG. **b** Proportion of Mo-MDSCs, G-MDSCs, Mφ, B cells, and T cells as percentage of CD45^+^ cells. **c** Number of CD45^+^ leukocytes per 100 μl of blood. Experiment repeated, five mice per group. ***P < 0.001, *P < 0.05; statistics: MC-TG versus saline. [MDSCs defined as CD11b^+^ MHCII^−^]

Since circulating MDSCs were restored 1 week post-TG injection, we sought to extend the MDSC-free window through multiple injections, namely on days 7 and 13 with 10 and 5 mg/kg MC-TG or free TG, respectively (Fig. 4a), doses that are cumulatively under the toxic threshold [43]. In both free and MC-TG-treated mice, Mo-MDSCs and G-MDSCs were reduced for approximately 10 days before starting to repopulate the blood by day 20 p.i. (Fig. 4b). As in Fig. 3, Mφ, B cells, and T cells were not affected by MC-TG or TG, as were total numbers of circulating CD45^+^ leukocytes (Fig. 4b, c).

**Fig. 5:**
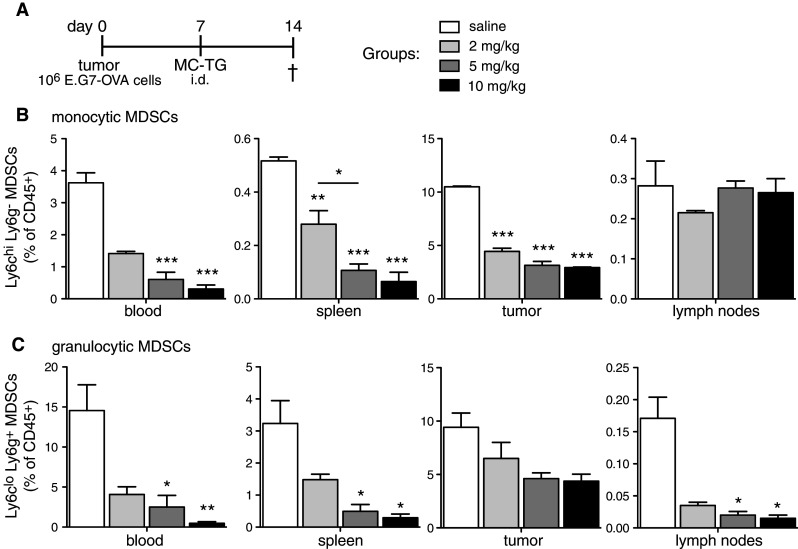
MDSC depletion is dose dependent. **a** Injection timeline of E.G7-OVA tumor-bearing mice injected 7 days p.i. with a solution of 2, 5, or 10 mg/kg MC-TG i.d. in the four footpads. Proportion of (**b**) Mo-MDSCs and of (**c**) G-MDSCs in the blood, spleen, tumor, and LNs on day 14 as percentage of CD45^+^ cells. Three mice per group, ***P < 0.001, **P < 0.01, *P < 0.05, statistics: MC-TG versus saline. [MDSCs defined as CD11b^+^ MHCII^−^]

Given the efficacy of the doses used above, we asked whether lower doses of MC-TG could provide similar efficacy. E.G7-OVA-bearing mice were injected with MC-TG 7 days p.i. with 2, 5, or 10 mg/kg (Fig. 5a). After 14 days, MC-TG-treated mice showed significantly reduced frequencies of Mo-MDSCs in the blood, spleen, and tumor, but not in LNs (Fig. 5b). In contrast, MC-TG led to a significant reduction in G-MDSCs levels in blood, spleen, and LNs, but not in the tumor (Fig. 5c). We observed a dose response to MC-TG, with the 10 mg/kg dose leading to a stronger reduction in MDSCs than the 5 mg/kg dose, which itself was more potent than the 2 mg/kg dose. These results show that two doses of MC-TG prolong MDSC depletion in the blood, that lower doses of MC-TG were effective at reducing MDSCs systemically and locally, and that the magnitude of MDSC depletion was dose dependent, with 10 mg/kg being the most effective dose.

### Depleting MDSCs with MC-TG enhances the efficacy of adoptive T cell therapy

We next asked whether MDSC depletion with MC-TG could improve the efficacy of a model cancer vaccine composed of OVA-conjugated and CpG-conjugated NPs [50]. E.G7-OVA-bearing mice were immunized 3 and 10 days p.i. with NP-OVA + NP-CpG and treated with 10 mg/kg MC-TG 13 days p.i. (supplementary Fig. S4 A). We chose this TG dose for its ability to deplete MDSCs systemically. After regressing, none of the tumors recurred in the mice receiving MC-TG, while 25 % of tumors recurred in immunized mice that did not receive MC-TG (supplementary Fig. S4 B). Five days post-MC-TG treatment, mice had almost a threefold reduction in OVA-specific CD8^+^ T cells compared to mice that did not receive MC-TG (supplementary Fig. S4 C). Immunized mice had reduced Mo-MDSC frequencies compared to control mice, and the addition of MC-TG rendered their levels undetectable by day 5 post-treatment (supplementary Fig. S4 D). MC-TG also reduced the frequencies of circulating G-MDSCs and Mφ compared to vaccine-only mice (supplementary Fig. S4 D).

**Fig. 6:**
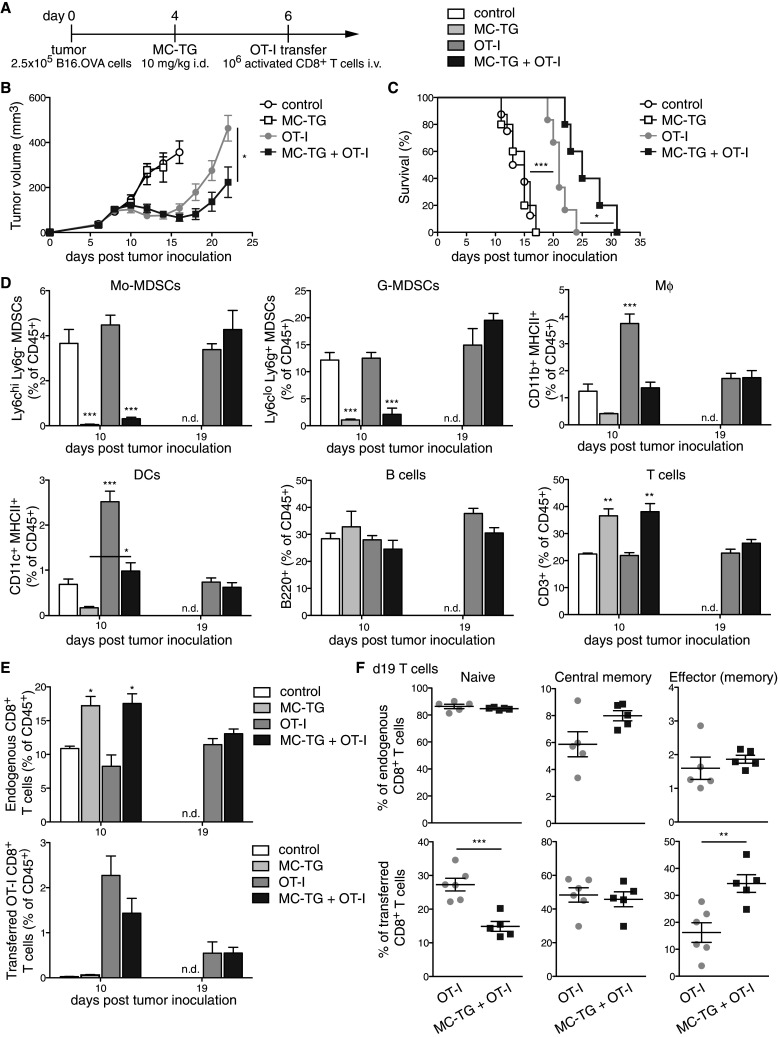
Depleting MDSCs with MC-TG enhances the efficacy of adoptive T cell therapy. **a** Injection timeline, **b** tumor volumes, and **c** survival of B16.OVA tumor-bearing mice injected with 10 mg/kg MC-TG i.d. in all four footpads 4 days p.i. and with 10^6^ activated OT-I T cells adoptively transferred i.v. 6 days p.i. **d** Proportion of Mo-MDSCs, G-MDSCs, Mφ, DCs, B cells, and T cells in the blood (as percentage of CD45^+^); statistics vs all other groups. **e** Proportions of endogenous and transferred OT-I T cells on days 10 and 19 p.i. (as percentage of CD45^+^). **f** Phenotype of *upper* endogenous CD8^+^ and *lower* transferred OT-I CD8^+^ T cells 19 days p.i. in the blood: naive (CD44^−^ CD62L^+^), central memory (CD44^+^ CD62L^+^), and effector (memory) (CD44^+^ CD62L^−^) T cells. 4–6 mice per group, ***P < 0.001, **P < 0.01, *P < 0.05, *n.d.* no data. [MDSCs defined as CD11b^+^ MHCII^−^]

Because MDSC depletion with MC-TG did not enhance anti-tumoral adaptive immunity to a vaccine targeted to tumor-draining LNs, we sought to assess potential benefits of depleting MDSCs in adoptive T cell therapy. Four days p.i., B16.OVA-bearing mice were injected with 10 mg/kg MC-TG, followed by adoptive T cell therapy on day 6 (Fig. 6a). B16.OVA tumors regressed 4 days after adoptive transfer (Fig. 6b). While MC-TG had no effect on B16.OVA tumor growth, a single dose of MC-TG prolonged tumor regression and delayed tumor growth in response to OT-I adoptive transfer, leading to significantly smaller tumors by day 22 p.i. (Fig. 6b). As a consequence, MC-TG + OT-I-treated mice demonstrated significantly enhanced survival compared to OT-I-treated mice without MDSC depletion (Fig. 6c). Free TG, on the other hand, did not affect tumor regression beyond the effect of OT-I transfer and did not enhance survival of tumor-bearing mice (supplementary Fig. S5 A–B). As in Fig. 3, MC-TG transiently reduced proportions of circulating Mo-MDSCs and G-MDSCs, as well as of Mφ and DCs, and all cell subsets repopulated the blood compartment 2 weeks post-MC-TG administration (day 19 p.i.) (Fig. 6d); free TG did not significantly reduce Mo- and G-MDSC levels (supplementary Fig. S5 C-D). B cells were not affected by MC-TG treatment, and proportions of T cells were higher in mice that received MC-TG compared to other groups (Fig. 6d). Consistent with this observation, frequencies of endogenous CD8^+^ T cells were more elevated in groups receiving MC-TG 10 days p.i., and no difference was observed between OT-I transferred mice 19 days p.i. (Fig. 6e). No differences in endogenous (non-OT-I) CD8^+^ T cell phenotype were detected (Fig. 6f *upper*). Among adoptively transferred mice, more OT-I CD8^+^ T cells had an effector (memory) and fewer a naïve phenotype in mice treated with MC-TG after 2 weeks (Fig. 6f *lower*). Together, these results show that MDSC depletion with MC-TG enhanced the anti-tumor efficacy of adoptively transferred OT-I CD8^+^ T cells, suggesting that MC-TG created a therapeutic period for transferred T cells to kill tumor cells by reducing the suppressive MDSCs in the tumor microenvironment. These results also confirm the therapeutic superiority of MC-TG over free TG in the context of adoptive T cell immunotherapy.

## Discussion

In this study, we explored the use of MC-TG as MDSC-targeting and MDSC-depleting agent in tumor-bearing mice. We found that MC-TG effectively depleted Mo-MDSCs in vitro and in vivo in two different tumor models. In tumor-bearing mice, MC-TG also targeted G-MDSCs and Ly6c^hi^ MΦ. Although targeting MDSCs with MC-TG did not enhance the efficacy of a model cancer vaccine, it enhanced therapeutic benefits of adoptive T cell therapy, suggesting that using MC-TG for efficiently depleting MDSCs may improve the efficacy of adoptively transferred anti-tumor T cell therapies.

We first demonstrated the efficacy of MC-TG in depleting BM-derived Mo-MDSCs (Fig. 1). This in vitro model has limitations in that cells are derived from naïve mice rather than from tumor-bearing mice. When the drug had good access to cells in vitro, obviating the need for targeted delivery, free TG was as effective as the micellar form, MC-TG. Therefore, we hypothesized that nanosized formulations become differentially effective only in vivo where biodistribution and transport, particularly in lymphatic drainage and LN targeting [53], are improved over the diffuse and broader distribution of the free drug [54].

For in vivo studies, we chose the E.G7-OVA thymoma model for its strong MDSC recruitment and accumulation [55]. MC-TG specifically depleted Mo-MDSCs in the spleen, G-MDSCs in LNs, and Mo-MDSCs in the tumor (Fig. 2). MC-TG depleted each MDSC subset in the relevant organ, making these findings very promising. Moreover, MC-TG targeted G-MDSCs in LNs, suggesting that MC-TG efficiently drained through lymphatics, consistent with our previous biodistribution study on similar NPs [47]. While MC-TG did not deplete G-MDSCs in vitro, we hypothesize that the time difference between in vitro and in vivo experiments (3 vs 5–7 days) may explain the discrepancy between in vitro and in vivo observations. We previously showed that nanoparticulate carriers could associate externally with B and T cells without being internalized [47], which may explain why B cells associated with MC-TG in tumors without being depleted (Fig. 2e); further work is, however, needed to determine why B cells associated with MC-TG in tumors but not in spleens or LNs. High doses (100–200 μg) of CpG have been reported to deplete tumor Mo-MDSCs after intratumoral injection and to deplete splenic Gr1^hi^ MDSCs after subcutaneous injection [36, 37]; however, this method required direct intratumoral injection to target tumor-infiltrating MDSCs, while we were able to target and deplete the same cells with indirect, passive i.d. delivery of MC-TG.

We next found that depletion of MDSCs in the blood peaked 7 days post-injection (Fig. 3) and that MC-TG was more effective at doing so than free TG. After being depleted, MDSCs repopulated the blood to finally surpass their control populations, suggesting a compensatory mechanism in hematopoiesis. We hypothesized that Mo-MDSCs were more readily depleted because they can further divide and proliferate, while G-MDSCs cannot [56]. These results were reproducible in the B16-F10 melanoma model, which also recruits significant MDSC numbers [55]. Similarly to what has been reported with RA [10], depleting MDSCs with MC-TG did not affect tumor growth (Figs. 3, 6), suggesting that MDSC depletion on its own did not sufficiently impact ongoing anti-tumor immunity. It has been shown that depletion of MDSCs with other drugs leads to delayed tumor growth, suggesting that MDSC depletion with MC-TG acts differently on E.G7-OVA and B16-F10 tumor growth than with other drugs [10, 11, 34, 35, 37]. While we aimed to specifically deplete MDSCs, the observation that MΦ were also targeted is not surprising given the use of TG and other thiopurine drugs as chemotherapeutics for myeloid and myelogenous leukemias, where monocyte and granulocyte precursors are targeted [42, 44].

Finally, we combined MC-TG treatment with two different modalities of cancer immunotherapy and found that our MDSC-depleting strategy enhanced adoptive T cell therapy and led to an enhanced effector phenotype of transferred OT-I CD8^+^ T cells (Fig. 6). This suggests that MC-TG created a period of time that enabled transferred T cells to infiltrate the tumor and kill tumor cells without being immune suppressed by the tumor microenvironment. Although other groups have reported that targeting MDSCs in combination with a cancer vaccine can improve immune outcomes [10, 11, 57], we found no therapeutic benefit in combining MC-TG treatment with a potent anti-tumor vaccine that delivered antigen and adjuvant to LNs (supplementary Fig. S4) [50]. The lack of response to a LN-targeting vaccine may be related to the transient reduction in other myeloid cells, namely MΦ and DCs, which may thus inhibit adaptive immunity to vaccination; this indeed correlated with a decrease in circulating OVA-specific CD8^+^ T cells. In contrast, the efficacy of adoptively transferred effector T cells, which do not require antigen presentation and priming steps, was enhanced when combined with MC-TG-mediated MDSC depletion (Fig. 6).

In summary, these data suggest that MC-TG can be used to efficiently target and deplete Mo-MDSCs and G-MDSCs, as well as monocytic MΦ. We further show that MC-TG was more efficacious than equivalent doses of free TG in depleting MDSCs in vivo, with a peak response after 7 days. When used in combination with adoptive transfer of activated, anti-tumor effector CD8^+^ T cells, MC-TG, but not free TG, could significantly improve therapeutic outcome by depleting suppressive MDSCs, thus allowing the T cells to be more effective in the tumor microenvironment.

### Electronic supplementary material

Below is the link to the electronic supplementary material.

Supplementary material 1 (PDF 463 kb)

## Abbreviations

BM: Bone marrow
DC: Dendritic cell
G (-MDSC): Granulocytic
i.d.: Intradermal(ly)
i.v.: Intravenous(ly)
LN: Lymph node
MC: Micelle
MDSC: Myeloid-derived suppressor cell
Mo (-MDSC): Monocytic
MΦ: Macrophage
NP: Nanoparticle
OVA: Ovalbumin
PEG: Poly(ethylene glycol)
PPS: Poly(propylene sulfide)
p.i.: Post-tumor inoculation
RA: Retinoic acid
RBC: Red blood cell
TG: 6-Thioguanine
