# Supplementary material for: 6-Thioguanine-loaded polymeric micelles deplete myeloid-derived suppressor cells and enhance the efficacy of T cell immunotherapy in tumor-bearing mice
Source: Cancer Immunol Immunother. 2015 May 16;64(8):1033–46. doi: 10.1007/s00262-015-1702-8 (PMC4506469; doi:10.1007/s00262-015-1702-8)
Supplement: Supplementary file 1 — Supplementary material 1 (PDF 463 kb) [file 262_2015_1702_MOESM1_ESM.pdf]

Supplementary material

Figure S1

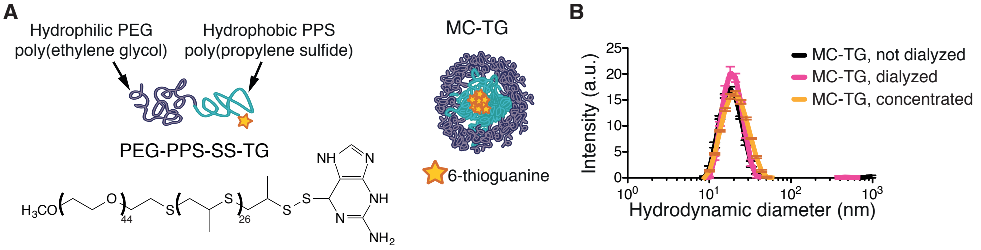

Figure S1. Formulation and characterization of MC-TG. (A) Structure and **(B)** characterization by DLS of 6-thioguanine micelles (MC-TG) at the different steps of purification.

Figure S2

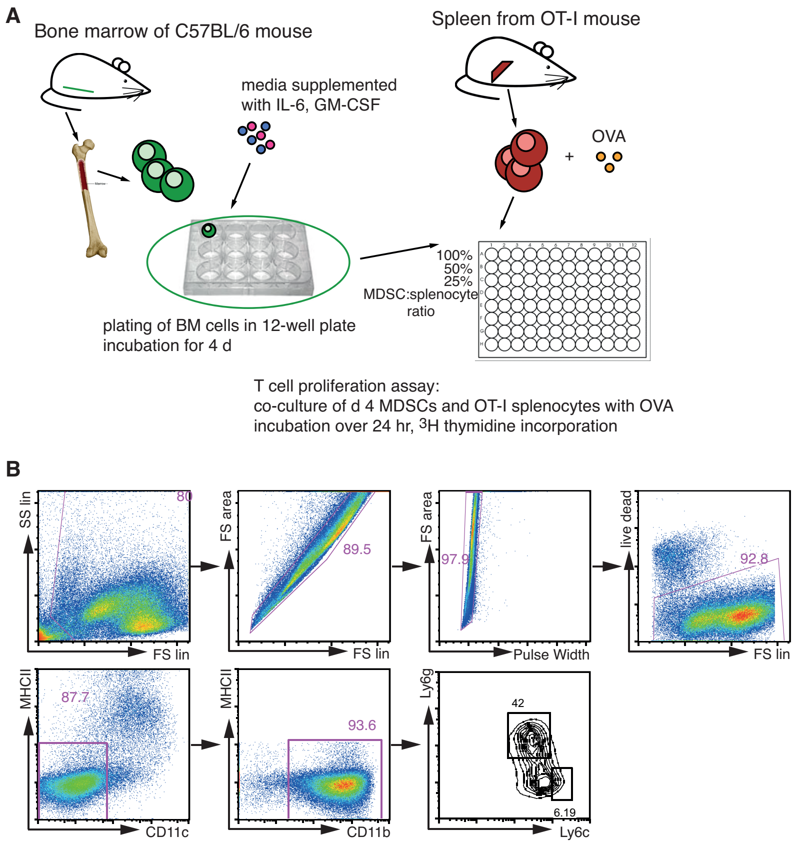

Figure S2. *In vitro* bone marrow culture and gating strategy. (A) Generation of MDSCs *in vitro* from bone marrow and MDSC co-culture with OT-I splenocytes and OVA. **(B)** Gating strategy of cells analyzed by flow cytometry: cells are selected by SS vs FS gating, singlets, live cells, MHCII$^-$ CD11c$^-$ gating, then CD11b$^+$, and finally Ly6c^{hi} and Ly6g$^+$ cells are analyzed.

Figure S3

E.G7-OVA lymphoma

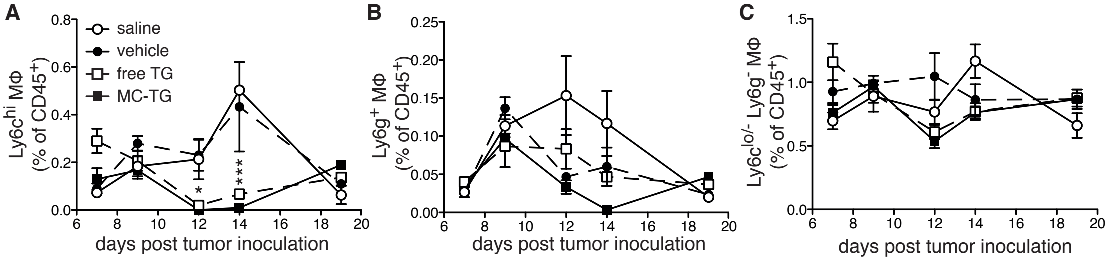

Figure S3. MC-TG depletes circulating monocytes in tumor-bearing mice.

Proportion of circulating **(A)** Ly6c^{hi} Mϕ, **(B)** Ly6g^{+} Mϕ, and **(C)** Ly6c$^{lo/-}$ Ly6g^{-} Mϕ over time as percentage of CD45^{+} cells in E.G7-OVA tumor-bearing mice injected 7 d p.i. with 10 mg/kg TG i.d. in the 4 footpads. Experiments repeated, 4-5 mice per group. ***P<0.001, **P<0.01, *P<0.05. [MDSCs defined as CD11b^{+} MHCII^{-}, Mϕ as CD11b^{+} MHCII^{+}, B cells as B220^{+}, T cells as CD3^{+}]

Figure S4

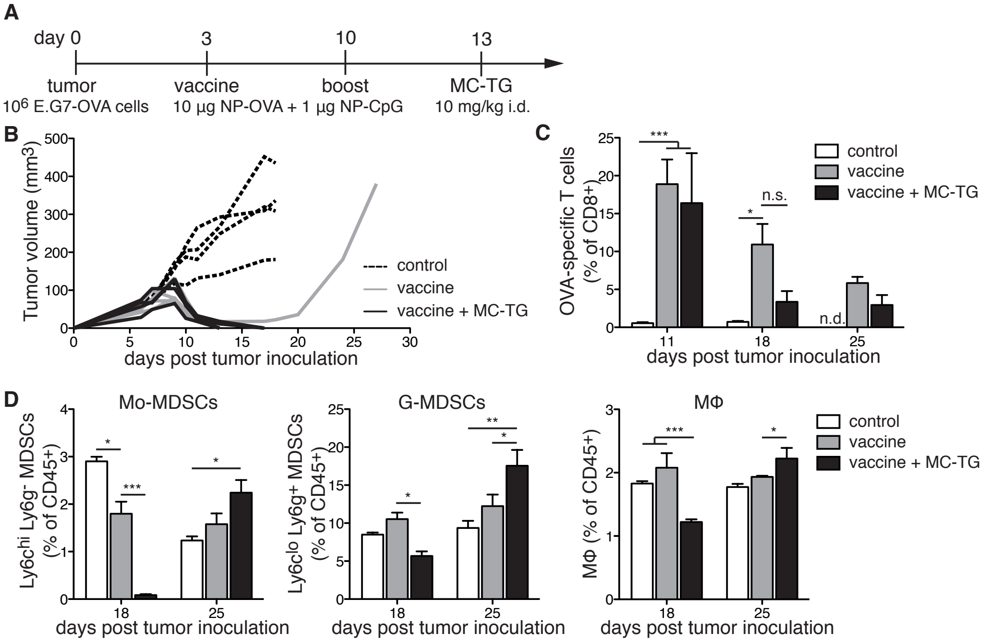

Figure S4. Depleting MDSCs does not increase the efficiency of a NP-based cancer vaccine in E.G7-OVA bearing mice. (A) Injection timeline of E.G7-OVA tumor-bearing mice immunized on d 3 and 10 with 10 µg NP-OVA + 1 µg NP-CpG i.d. in the front footpad and injected 7 d p.i. with 10 mg/kg MC-TG i.d. **(B)** E.G7-OVA tumor volumes of each individual mouse. **(C)** Proportion of OVA-specific $CD8^+$ T cells as determined by SIINFEKL-MHCI pentamer staining. **(D)** Proportion of Mo-MDSCs, G-MDSCs, and Mϕ as percentage of $CD45^+$ cells. 4 mice per group, ***P<0.001, **P<0.01, *P<0.05, n.s. not significant, n.d. no data. [MDSCs defined as $CD11b^+$ $MHCII^-$]

Figure S5

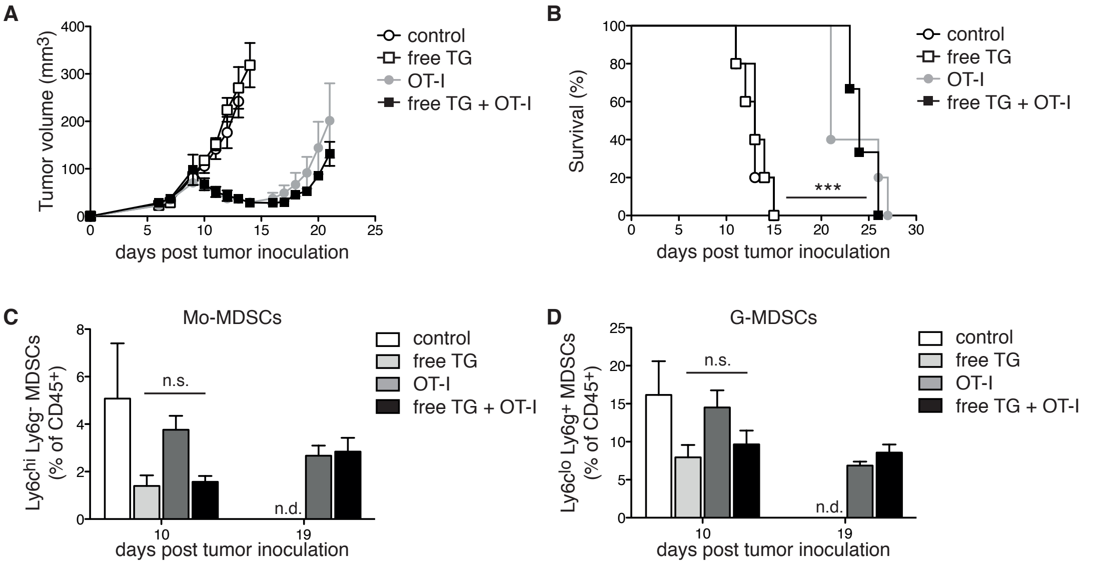

Figure S5. Free TG does not enhance the efficacy of adoptive T cell therapy. (A) Tumor volumes and **(B)** survival of B16.OVA tumor-bearing mice injected with 10 mg/kg free TG i.d. in all 4 footpads 4 d p.i. and with 10^6 activated OT-I T cells adoptively transferred i.v. 6 d p.i.. Proportion of **(C)** Mo-MDSCs and **(D)** G-MDSCs in the blood (as percentage of $CD45^+$). 4-6 mice per group, ***P<0.001, n.s. not significant, n.d. no data. [MDSCs defined as $CD11b^+$ $MHCII^-$]
